# 1H NMR-based dynamic metabolomics delineates the therapeutic effects of Shenfu injection on laparoscopic hysterectomy

**DOI:** 10.1097/MD.0000000000023336

**Published:** 2020-12-24

**Authors:** Xue Wang, Kai Wang, Huan Wang, Xiangkui Li, Jinzhou Feng, Qian Chen

**Affiliations:** a Department of Anesthesiology, East Hospital of Sichuan Provincial People's Hospital; b Department of Acute Care Surgery, Sichuan Provincial People's Hospital, Sichuan Academy of Medical Sciences; c Department of Neurosurgery, East Hospital of Sichuan Provincial People's Hospital, Chengdu, China.

**Keywords:** hysterectomy, injections, laparoscopes, metabolomics, proton magnetic resonance spectroscopy

## Abstract

To explore the effect and mechanism of Shenfu Injection on serum metabolomics in laparoscopic hysterectomy.

1.5 mL/kg Shenfu injection was added to inject 200 mL of normal saline after the patients who entered the standard were admitted to the operating room. NMR metabolomics were performed at each time point before anesthesia (T0), immediately after pneumoperitoneum (T1), and at the end of surgery (T2).

Multivariate trajectory analysis showed that SFI treatment could make laparoscopic hysterectomy interfere with the recovery of plasma metabolites to normal metabolic state, with a time-dependent trend. In addition, the key metabolic changes of laparoscopic hysterectomy at different stages of SFI treatment involve energy metabolism, oxidative stress response, amino acid metabolism, and pyruvate metabolism. Especially, the important role of SFI in the treatment of laparoscopic hysterectomy is antioxidant capacity. The results show that SFI can be used as a potential drug for laparoscopic hysterectomy.

The current findings provided, for the first time, sound evidence of the protective effects of SFI on laparoscopic hysterectomy from both biochemical and metabolomics perspectives. The mechanisms of SFI could be related to regulating amino acid metabolism, pyruvate metabolism, and energy metabolism. The present study lays an important foundation for further research and for the broad clinical application of SFI.

## 1 Introduction

Hysterectomy is one of the most commonly performed gynaecological surgery procedures.^[1]^ The widespread use of endoscopic surgery leads to increasing laparoscopic hysterectomy uses, with the incidence rates of 12% in United States.^[2]^ Laparoscopic surgery has the characteristics of small trauma, rapid recovery, and less complication. However, carbon dioxide pneumoperitoneum can cause a series of pathophysiological changes, including stress response, respiratory, circulatory, and endocrine system changes.

Studies have shown that carbon dioxide pneumoperitoneum pressure, duration, anesthesia, anesthetics, and other related to the pathophysiological changes during surgery, pneumoperitoneum can cause stress response, because of the pneumoperitoneum elevation, diaphragmatic movement, restricted movement, chest lung compliance, causing changes in the microenvironment of the chest and abdomen, making the body sympathetic-adrenal medullary system excited, releasing catecholamines, vasopressin, kidney Sustained, such as increased heart rate, elevated blood pressure. The stress response not only causes a large release of cortisol (COR), but also increases blood sugar, which will also lead to changes in cytokine concentration.

Traditional Chinese Medicine (TCM) has been practiced in China for thousands of years and is becoming more and more popular all over the world to improve human health, especially the treatment of chronic diseases.^[3]^ Nowadays, various combinations of traditional Chinese medicine have shown their unique advantages in the clinical treatment of cardiovascular disease (CVD). Shenfu Injection (SFI) is a classical Chinese medicine formulation (TCMF), which has a long history of using to alleviate oxidative stress in China. It consists of Astragalus membranaceus, ginseng, licorice processing and cinnamon. Many active ingredients (such as ginsenoside, astragaloside, glycyrrhizin, flavonoids, etc) have been identified from SFI, and have shown some effects in alleviating stress during general anesthesia induction and tracheal intubation in previous studies.^[4–6]^ SFI has been used in various laparoscopic surgeries, including laparoscopic cholecystectomy, laparoscopic myomectomy, and laparoscopic splenectomy,^[7–9]^ however, there is no research focused on the effect of SFI on laparoscopic hysterectomy. Still the mechanism of SFI treatment of laparoscopic hysterectomy remains unclear.

Metabonomics is a new discipline developed in the late 1990s. It is a science that quantitatively describes the endogenous metabolites of organisms and their responses to internal and external factors.^[10]^ Nuclear magnetic resonance (NMR) can be used for both qualitative analysis of mixed systems and for quantitative analysis. In the past few decades, with the rapid development of analytical techniques and various experimental techniques, quantitative methods based on nuclear magnetic resonance have been widely used in the analysis of biological samples. NMR quantitative analysis technology is applied to metabolomics and has become an important means in quantitative metabolomics research. Traditional Chinese medicine is a gem of the Chinese nation, a crystallization of the sublimation of the philosophical view of simple materialism and the accumulation of thousands of years of clinical experience. However, the methods and theories of Chinese medicine for understanding diseases lack a suitable modern scientific representation system. Therefore, establishing a bridge between the two scientific systems of Chinese medicine and modern medicine has become an important proposition in the field of modern life sciences. The rise and development of systems biology, especially metabolomics, provides an opportunity and challenge for the modernization of Chinese medicine, and serves as a bridge between Chinese medicine and modern medicine research.^[11]^

In this study, 40 patients with ASA (I–II) were undergone laparoscopic hysterectomy, and half of them were administrated SFI. Then, a dynamic metabolomic trajectory analysis based on proton magnetic resonance (1H NMR) combined with pharmacodynamics was first proposed to study the pathological process of laparoscopic hysterectomy and evaluate the potential protective effect and mechanism of SFI on laparoscopic oxidative stress during hysterectomy.

## 2 Methods

### 2.1 Patients’ characteristics

All participants were treatment-naïve and enrolled from East Hospital of Sichuan Provincial People's Hospital with written informed consent. The protocol of the study was approved by Institutional Review Board of East Hospital of Sichuan Provincial People's Hospital. All experiments were conducted in accordance with the approved guideline, which was approved by the Ethics committee of Sichuan Provincial People's Hospital (2019 No. 254).

Study group (SFI group): After the patients who entered the standard were admitted to the operating room, the ECG monitor was monitored, and the subclavian vein catheter was placed in parallel. Then, 1.5 mL/kg Shenfu injection was added to inject 200 mL of normal saline. After the instillation was completed, anesthesia induction was performed.

**Figure 1:**
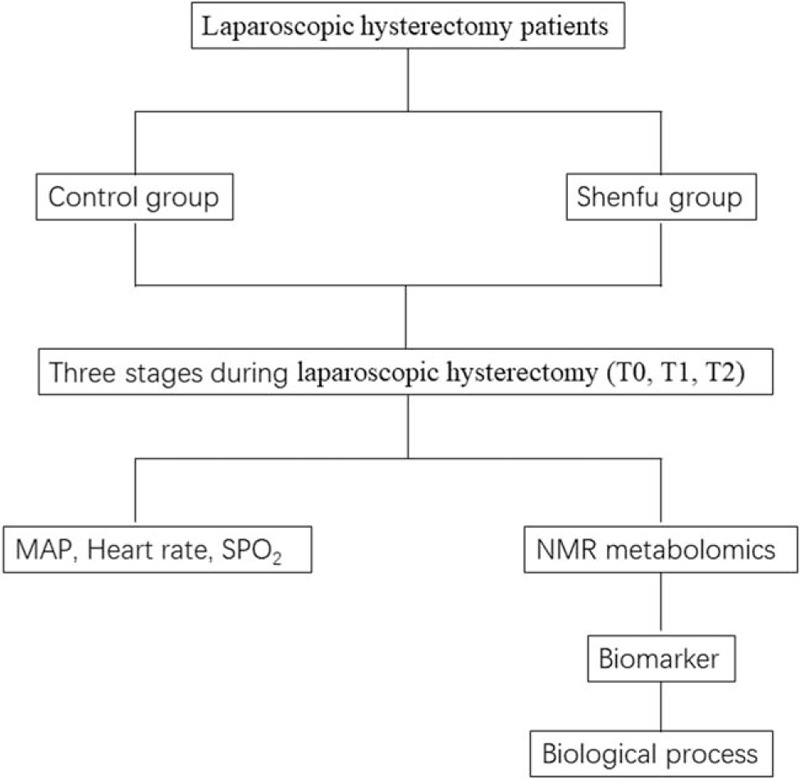
Flow diagram of study protocol.

Control group: After the patients who entered the standard were admitted to the operating room, the ECG monitor was monitored, and the subclavian vein catheter was placed in parallel, followed by anesthesia induction and surgery. The mean arterial pressure (MAP), heart rate (P), and pulse oximetry (SPO2) at each time point before anesthesia (T0), immediately after pneumoperitoneum (T1), and at the end of surgery (T2) were recorded (Fig. 1).

### 2.2 Inclusion and exclusion criteria

Laparoscopic surgery has the characteristics of small trauma, rapid recovery, and less complications. However, carbon dioxide pneumoperitoneum can cause a series of pathophysiological changes, causing a strong stress response in the body, leading to obvious changes of respiratory, circulatory and endocrine systems. In this study, 40 patients with ASA (I–II) were included, whose ages range from 30 to 65. 1. The patients have severe heart, high blood pressure, diabetes, respiratory insufficiency, asthma, and other medical history were excluded. All patients signed the consent of this surgery.

### 2.3 Drugs and reagents

Shenfu injection (SFI, lot. Number 150725010) was donated by YaAn Sanjiu Pharmaceutical Co, Ltd (Sichuan, China). The main components of SFI include ginsenosides (>0.8 mg/mL) and aconitine (<0.1 mg/mL).

### 2.4 Preparation of plasma samples and 1H NMR spectroscopy

For plasma, 200 μL of the melted plasma sample was mixed with 400 μL of saline solution (pH 7.4, 0.9% NaCl, 50% D_2_O, and 0.1% TSP); the mixture was homogenized by eddy current for 30 s and centrifuged at 12,000 rpm at 4°C for 10 min. A homogeneous sample of each supernatant was then transferred to a 5 mm NMR tube. Plasma quality control (QC) samples were prepared by mixing equal volume (10 μL) samples from each sample and using the same procedure described above. Before analysis, all samples were stored at 4°C. The collected QC samples were used to optimize the 1H NMR spectral parameters, and then the optimized spectral acquisition parameters for each sample analysis were used.

All NMR spectra were obtained on a 500 MHz NMR spectrometer system (Varian, Palo Alto, CA) at 300 K. The 90 degree pulse length is adjusted to 10 s. The one-dimensional (1D) 1H NMR spectra of each sample were recorded by using the NOESY pulse sequence to better understand all types of molecules with a relaxation delay of 4S and a mixing time of 100 ms. In the case of 16 ppm spectrum width, 64 transients were collected into 32 k data points. Plasma samples were analyzed by 1D 1H NMR Carr Purcell meiboom gill (CPMG) pulse sequence to attenuate macromolecular signals (proteins and lipids). During 4S relaxation delay and 80 ms mixing time (32 k data points; 16 ppm spectral width), 128 transient signals were collected by radiation. All 1H NMR spectra of manually phased plasma samples from mestrelab research s.l. in Spain were used and TSP (10.0) was baseline calibrated. According to the human metabonomics database (HMDB; http://www.hmdb.ca/), the Madison metabonomics joint database (mmcd; http://mmcd.nmrfam.wisc.Edu/), the biomagnetic resonance database allocates resonance (BMRB; http://www.bmrb.wisc.edu), and public data or references on chemical shifts.

### 2.5 Data processing for metabolomic analysis

Two different data processing methods, including isometric classification and combination of “normalized to total spectrum area” (NTSA), and integration combination of adaptive intelligence (AI) and probability quotient normalization (pnq), are used to obtain NMR matrix data. Remove the δ_H_ 4.67 to 5.20 region of plasma sample to eliminate the influence of water unsaturated. One dimensional spectral region (δ^H^ 0.50 to 7.00 for plasma samples) is integrated into a box with a smaller equidistant width (0.002 ppm for plasma samples); these data are then standardized using mest Renova's normalized to total spectral region (NTSA) method. In addition, the AI merging and pnq methods of mv-ack toolbox can also be used to analyze 1H NMR spectral data (http://bionmr.unl.edu/mvapack.php). Then, 1H NMR data matrix was submitted to multivariate statistical analysis (MVA) by Simca-p software (v14.1, umetric, ume å, Sweden). The first step is to use unsupervised principal component analysis (PCA) to assess quality and homogeneity, and to identify subgroups in the dataset. In addition, the supervised orthogonal partial least squares discriminant analysis (OPLS-DA) was used to classify and identify different metabolites. The quality of the MVA model is controlled by evaluating the cross validation parameters *R*^2^ and *Q*^2^. For the data processed by NTSA, the S-line load map with color coding is created by using the auto scaling OPLS-DA model, and the differentiated metabolites are presented as positive and negative signals by peak integration. Warm (e.g., red) signals significantly promote the differentiation of populations, and have a higher correlation than cold (e.g., blue) signals. In addition, the *P* value of CV-ANOVA is used to test the validity of the fitted OPLS-DA model. For the data obtained from AI sub boxes and pnq, we use S-graph and projection (VIP) graph of OPLS-DA model to analyze the influence of variables and Pareto scaling method to identify the highest potential variables. Data set. When the minimum absolute correlation coefficients (ABS P [CORR] and VIP values) also meet the statistical requirements of students not to test, they are used as statistical cut-off values (*P* < .05).

### 2.6 Statistical analysis

All the data were expressed as mean (+SD) and analyzed by SPSS17.0 statistical software. Single factor analysis of variance (ANOVA) was used between groups, and then Turkey's multiple comparison test was conducted. The value of *P* < .05 is considered to be statistically significant.

## 3 Results

### 3.1 SFI ameliorates MAP, Heart rate, SPO_2_, COR, GLU in laparoscopic hysterectomy

Table 1 shows the mean MAP, Heart rate, SPO_2_, COR, GLU at different time points in each group. However, there was no difference in MAP, heart rhythm, SPO2, COR, GLU between groups T0, T1, and T2.

**Table 1:** The effect of SFI on laparoscopic hysterectomy during the course of surgery.

	T0		T1		T2	
	SFI	Control	SFI	Control	SFI	Control
MAP	96.67 ± 11.67	91.0 ± 0.27	91 ± 4.10	92 ± 0.91	81.17 ± 6.31	83.3 ± 0.81
Heart rate	80.67 ± 16.29	69.17 ± 0.24	63 ± 8.90	59.8 ± 0.65	62.8 ± 9.02	63.2 ± 0.96
SPO_2_	98.83 ± 0.98	98.83 ± 1.00	100 ± 0.00	100 ± 0.00	100 ± 0.00	99 ± 0.36
COR	12.67 ± 3.92	8.58 ± 0.01	10.96 ± 4.81	5.46 ± 0.02	6.88 ± 4.24	3.44 ± 0.05
GLU	6.27 ± 1.34	5.45 ± 0.21	4.9 ± 0.66	5.28 ± 0.50	5.82 ± 1.12	6.6 ± 0.47

### 3.2 Quality assessment of 1H NMR measurement and metabolite identification

**Figure 2:**
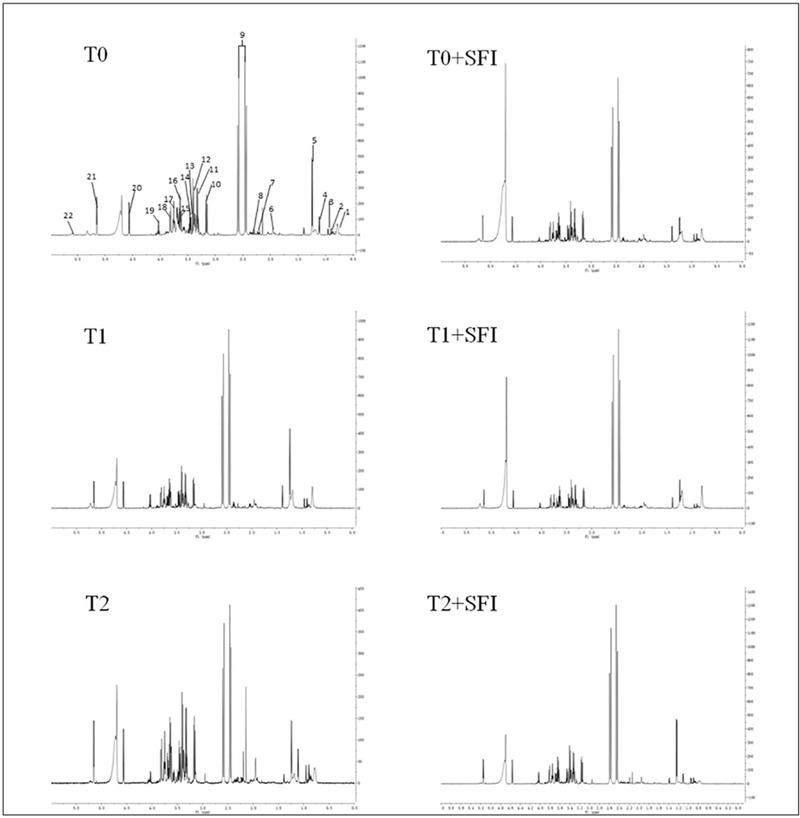
Representative 1H NMR spectra of plasma samples from various groups. (A) 1H NMR NOESY spectra of laparoscopic hysterectomy samples from different groups. (B) 1H NMR NOESY spectra of SFI treatment samples from different groups. Signal assignment: 1, 1, 2-hydroxyisovalerate; 2, leucine; 3, proline; 4, ethanol; 5, alanine; 6, acetate; 7, pyruvate; 8, succinate; 9, creatine; 10, phosphatidylcholine; 11, glucose; 12, choline; 13, citrate; 14, glutamate; 15, creatinine; 16, formate; 17, allantoin; 18, valine; 19, glutamine; 20, ethanolamine; 21, trigonelline; 22, glycine.

In this study, the methodologies for NMR-based metabonomic analysis were evaluated as follows. Six QC samples were analyzed by 1H NMR to evaluate the repeatability of 1H NMR measurements before actual sample collection. The relative standard deviation (RSD) of the five selected peaks is <2.5% and the peak displacement is <0.5%. In addition, 1H NMR spectra of QC samples were detected in 10 actual samples during the whole operation process to verify the chemical scheme, monitor the reliability and stability of the NMR operation system and correct measurement errors. The results of PCA score map show that QC samples are compactly clustered, and the RSD of five selected peak areas is <5% for normalized peak intensity and <1% for peak offset in spectral data of QC samples. These results indicate that the proposed 1H NMR method is robust and repeatable for metabolomic analysis. According to the chemical shifts published in the 1D NMR spectra database and literature, the main endogenous metabolites were identified and annotated in the typical 1H NMR NOESY spectra of plasma and 1H NMR spectra of plasma (Fig. 2). The main limitation of this method is that metabolites bound to plasma/serum proteins are not visible or significantly reduced in the obtained NMR spectra. To avoid this harmful effect, all plasma samples were analyzed based on relative 1H NMR peak intensity. After standard intensity data preprocessing (baseline correction, comparison, grading, normalization, and scaling transformation) of sample 1H NMR spectra, unsupervised and supervised MVA analysis is used to analyze NMR data.

### 3.3 SFI dynamically regulates metabolic fingerprints associated with laparoscopic hysterectomy

**Figure 3:**
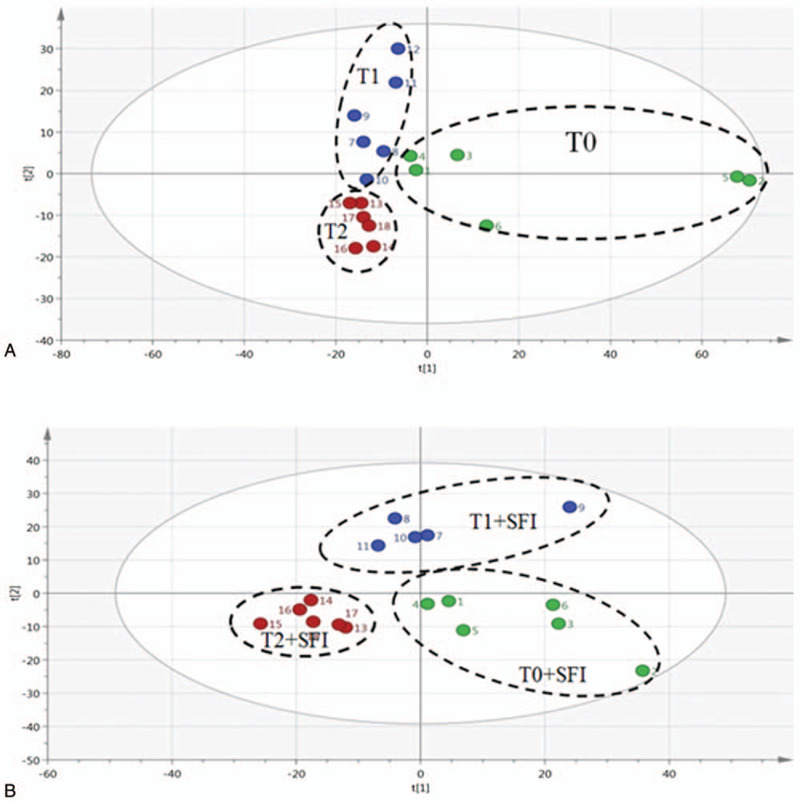
Trajectory derived from PCA score plot for assessing the effects of SFI on metabolic profiles of 1H NMR spectra of plasma samples from laparoscopic hysterectomy-induced CH rats. (A) PCA score plot of plasma samples from various laparoscopic hysterectomy groups. (B) PCA score plot of plasma samples from SFI treatment groups.

The dynamic trajectory analysis based on PCA was constructed for the first time to investigate the plasma and urine metabolites spectrum analysis at different time points in the operation and SIF groups, and to study the potential therapeutic effect of SFI on oxidative stress induced by laparoscopic hysterectomy. The PCA score map of dynamic trajectory was established, and the classification results were satisfactory (*R*^2^ = 0.82, *Q*^2^ = 0.62 in laparoscopic hysterectomy; *R*^2^ = 0.83, *Q*^2^ = 0.66 in SFI treatment), as shown in Figure 3. The spots in group T0 (laparoscopic hysterectomy) were separated from those in group T1 and T2, indicating the storage of plasma metabolic spectrum during laparoscopic hysterectomy. There were significant differences in plasma speckles, and the speckles were aggregated in T0, T1, and T2 SI groups respectively. However, the spots treated with SFI cannot be separated from those treated with laparoscopic hysterectomy. Overall, MVA results showed that SFI treatment significantly reversed the disturbed metabolic status of hysterectomy.

### 3.4 1H-NMR analysis and metabolic profiling for SFI treatment at T1

**Figure 4:**
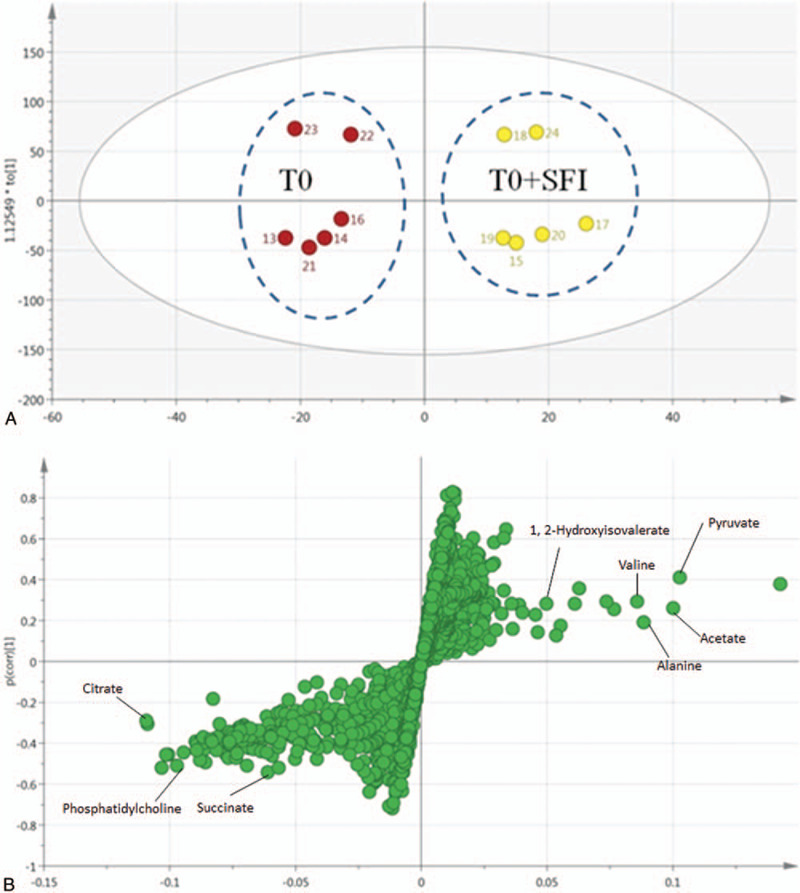
Score plot derived from the 1H-NMR spectra of serum from laparoscopic hysterectomy at T0 by PLS-DA analysis (A). S-plot (B) generated by comparison of metabolic profiles between the control and model groups based on PLS-DA.

Although the spots treated with SFI could not be separated from those treated with T0 and T2 laparoscopic hysterectomy, the spots at T1 gathered separately between SFI and laparoscopic hysterectomy. Prior to metabolic analysis, the original data were centered on mean and pareto-scaled. The metabolic patterns of four groups were analyzed by partial least squares discriminant analysis (PLS-DA), and it proved that the metabolic characteristics of T1 in laparoscopic hysterectomy were significantly different from those in SFI treatment group (Fig. 4). To prevent over-fitting of laparoscopic hysterectomy, the performance of PLS-DA model between the two groups was evaluated by 7-fold cross-validation and 200 replacements. In addition, the established supervised PLS-DA model shows good stability and prediction (*R*^2^*X* = 0.679, *R*^2^*Y* = 0.962, *Q*^2^ = 0.773). The results show that the model has good discrimination, adaptability and predictability.

**Figure 5:**
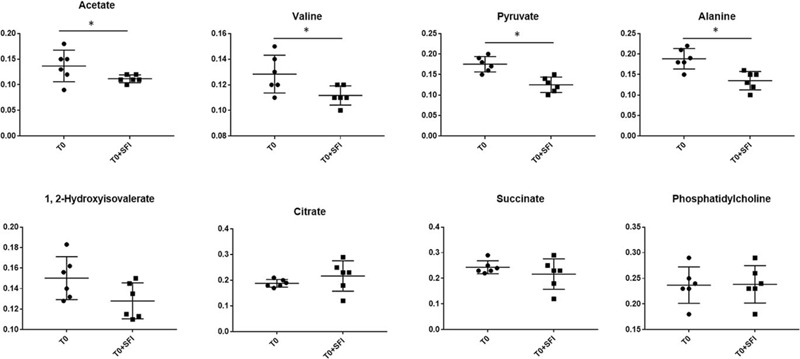
Box-plots of the relative intensities of potential biomarkers in different groups. Values were expressed as the means ± SD. ∗*P* < .05, ∗∗*P* < .01 compared with T0 group.

In the corresponding S plot, potential biomarkers were identified, indicating the importance of each variable to the classification (Fig. 4B). Based on the cutoff standards with VIP values (>1) and *P* values (<0.05), 8 metabolites were screened out as potential biomarkers. We found that the urinary levels of alanine, 1, 2-hydroxyisovalerate, valine, pyruvate, acetate were significantly increased, while the levels of phosphatidylcholine, succinate and citrate were significantly decreased in the SFI group compared with that in the T0 group. To assess the effects of SFI on T0 patients, the relative intensities of 8 potential metabolites were calculated using peak intensities from the resultant data matrices. The box plots (Fig. 5) showed changes in discriminatory metabolites in response to the effects of SFI on these changes.

### 3.5 Metabolic pathways analysis

**Figure 6:**
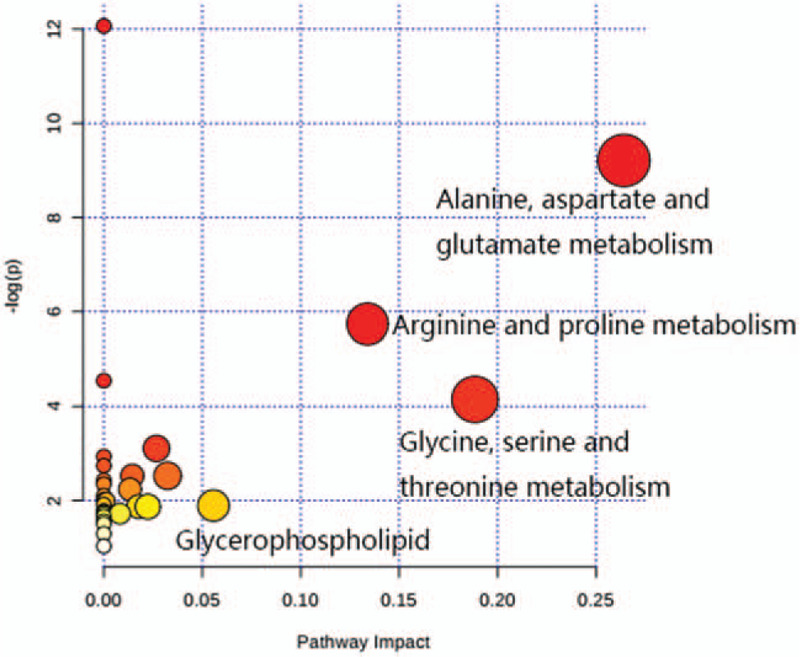
Summary of pathway analysis with MetaboAnalyst 3.5. Each point represents one metabolic pathway; the size of the dot and shades of color are in a positive correlation with the impact of the metabolic pathway.

**Figure 7:**
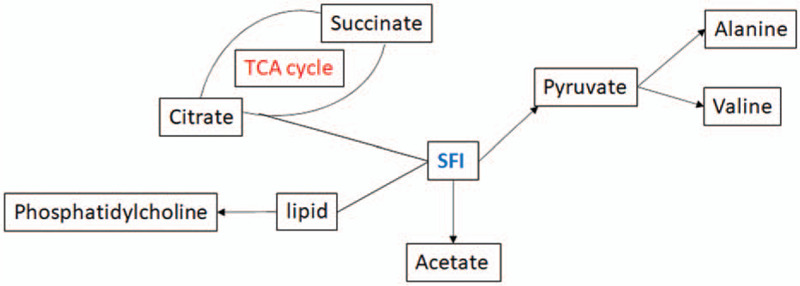
Schematic diagram of the disturbed metabolic pathway in response to laparoscopic hysterectomy. The network of the identified key biomarkers and pathways was constructed according to the KEGG pathway database.

Metabo Analysis 3.5 was used to analyze the pathways of 8 biomarkers. As shown in Figure 6, the biosynthesis/degradation of valine, leucine and isoleucine, arginine and proline metabolism and pyruvate metabolism are the most affected metabolic pathways associated with laparoscopic hysterectomy. Additionally, a network of the identified key biomarkers and related metabolic pathways in response to laparoscopic hysterectomy was constructed (Fig. 7).

## 4 Discussion

In the past 30 years, laparoscopic examination has become an important tool for the diagnosis and treatment of various gynecological diseases. Compared with laparotomy, its recognized advantages have ignited the worldwide application of laparoscopic surgery in the main selection of surgical methods under benign and malignant conditions.^[12]^ Laparoscopy is currently the first choice in the diagnosis and treatment of infertility related problems, such as endometriosis, leiomyoma, ovarian and fallopian tube lesions.^[13,14]^ However, it has been questioned whether carbon dioxide (CO_2_) pneumoperitoneum in laparoscopy itself can cause ovarian damage and endanger reproductive potential due to ischemia-reperfusion injury. In previous studies, it was reported that increased intra-abdominal pressure (IAP) after CO_2_ blowing reduced blood flow to solid organs by 10% to 80% during laparoscopic examination, and normalized by intra-abdominal inflation.^[15]^ In terms of perfusion insufficiency and reperfusion, these hemodynamic changes represent a typical model of ischemia-reperfusion, which leads to the production of reactive oxygen species (ROS). Because of the influence of ROS on cell injury and death, the over production of ROS is widely considered to be the main cause of several kinds of pathology. Although in some laboratory animal studies, even at safe IAP levels, CO2 pneumoperitoneum has been shown to be associated with severe ovarian damage caused by oxidative stress,^[16–18]^ there has been no clinical investigation showing its effects in human ovaries.

The lack of oxygen and nutrients in the blood during ischemia creates such conditions, in which the recovery of circulation leads to inflammation and oxidative damage by inducing oxidative stress rather than restoring normal function. In recent years, TCM has made great progress in the treatment of ischemic cardiovascular disease. SFI has been widely used in the treatment of heart failure, arrhythmia, acute myocardial infarction, dilated cardiomyopathy, cor pulmonale, and other cardiovascular diseases. Since SFI was approved by China food and Drug Administration (CFDA) and applied in many hospitals and clinics, it has produced obvious curative effect on patients.

These metabonomics studies enable us to identify potential differential metabolites to understand the mechanism of oxidative stress induced by laparoscopic hysterectomy and the therapeutic mechanism of SFI. Dynamic metabonomics analysis showed that SFI can recover the characteristics of plasma metabolites disturbed by hysterectomy, which is characterized by a time-dependent normal metabolic state. Supervised multivariate analysis showed changes in urine and plasma metabolites related to SFI. A group of metabolites used to distinguish between hysterectomy and different stages of SFI reflected the potential ability of SFI to improve the metabolic disorder caused by hysterectomy, which was mainly related to energy metabolism and oxidation, amino acid metabolism and intestinal microbial metabolism.

## Author contributions

**Conceptualization:** Xue Wang, Qian Chen.

**Data curation:** Xue Wang, Kai Wang, Huan Wang, Xiangkui Li.

**Writing – original draft:** Xue Wang, Qian Chen.

**Writing – review & editing:** Kai Wang, Huan Wang, Xiangkui Li, Jinzhou Feng, Qian Chen.
